# Leaf Damage Based Phenotyping Technique and Its Validation Against Fall Armyworm, *Spodoptera frugiperda* (J. E. Smith), in Maize

**DOI:** 10.3389/fpls.2022.906207

**Published:** 2022-07-11

**Authors:** P. Lakshmi Soujanya, J. C. Sekhar, K. R. Yathish, Chikkappa G. Karjagi, K. Sankara Rao, S. B. Suby, S. L. Jat, Bhupender Kumar, Krishan Kumar, Jyothilakshmi Vadessery, K. Subaharan, Jagadish Patil, Vinay K. Kalia, A. Dhandapani, Sujay Rakshit

**Affiliations:** ^1^Winter Nursery Centre, ICAR-Indian Institute of Maize Research, Hyderabad, India; ^2^Unit Office, ICAR-Indian Institute of Maize Research, New Delhi, India; ^3^DBT-National Institute of Plant Genome Research, New Delhi, India; ^4^ICAR-National Bureau of Agricultural Insect Resources, Bengaluru, India; ^5^ICAR-Indian Agricultural Research Institute, New Delhi, India; ^6^ICAR-National Academy of Agricultural Research Management, Hyderabad, India; ^7^ICAR-Indian Institute of Maize Research, Punjab Agricultural University, Ludhiana, India

**Keywords:** maize germplasm, invasive insect pest, fall armyworm, artificial infestation, leaf damage rating, phenotyping technique, resistant genotypes, injury levels

## Abstract

Globally, maize is an important cereal food crop with the highest production and productivity. Among the biotic constraints that limit the productivity of maize, the recent invasion of fall armyworm (FAW) in India is a concern. The first line of strategy available for FAW management is to evaluate and exploit resistant genotypes for inclusion in an IPM schedule. Screening for resistant maize genotypes against FAW is in its infancy in India, considering its recent occurrence in the country. The present work attempts to optimize screening techniques suited to Indian conditions, which involve the description of leaf damage rating (LDR) by comparing injury levels among maize genotypes and to validate the result obtained from the optimized screening technique by identification of lines potentially resistant to FAW under artificial infestation. Exposure to 20 neonate FAW larvae at the V_5_ phenological stage coupled with the adoption of LDR on a 1–9 scale aided in preliminary characterize maize genotypes as potentially resistant, moderately resistant, and susceptible. The LDR varies with genotype, neonate counts, and days after infestation. The genotypes, *viz*., DMRE 63, DML-163-1, CML 71, CML 141, CML 337, CML 346, and wild ancestor *Zea mays* ssp. *parviglumis* recorded lower LDR ratings against FAW and can be exploited for resistance breeding in maize.

## Introduction

Maize (*Zea mays* L.), the third most important cereal food crop after wheat and rice, is grown over 197 million ha across 170 countries with a total production of 1.148 billion MT (FAOSTAT, 2019). India ranks 4th and 7th in the world concerning area and production, respectively. In India, maize is grown in an area of 9.2 million ha with a production of 31.51 million MT (DES, 2021). It is widely used as animal and poultry feed, human food, and also serves as raw material in several industries like starch, food processing, pharmaceuticals, textile, and so on (Rakshit and Chikkappa, 2018; Choudhary et al., 2020). Several factors which affect maize productivity are biotic and abiotic stresses (Prasanna et al., 2021; Shemi et al., 2021), low farm mechanization, inadequate plant health management, and socio-economic conditions (Shiferaw et al., 2011). Among various biotic stresses, insect pests are one of the major factors responsible for low productivity. Globally, 90 different insect pests are reported to infest maize (Steffey et al., 1999). Among these, fall armyworm (FAW), *Spodoptera frugiperda* (J. E. Smith) (Lepidoptera: Noctuidae), native to tropical and sub-tropical regions of America (Rukundo et al., 2020), was one of the major insect pests for over a century in the Americas (Luginbill, 1928). However, it was not reported in other parts of the world until recent years when it spread to newer areas due to its highly migratory nature (Westbrook et al., 2016; Gonçalves et al., 2020) and come up as a major transboundary pest across maize-growing regions of the world. The rapid spread of FAW to Africa and Asian countries, including India, in recent years, is a threat to global food security (Daudi et al., 2021). The outbreak of FAW in central and western Africa was first reported in 2016 followed by Tanzania (January 2017), Kenya (April 2017), and Uganda (May 2017) (De Groote et al., 2020); by 2018, it has invaded Sub-Saharan Africa, Sudan [FAO (Food and Agriculture Organization of the United Nations), 2018], Middle East (Yemen) (FAO, 2019a), and the Indian sub-continent. In India, FAW was first reported in May 2018 in Karnataka (Sharanabasappa et al., 2018; Shylesha et al., 2018). Since its first report, it has spread across the country within a year (Rakshit et al., 2019) and started infesting maize leading to significant economic damage in all maize-growing areas. FAW continued to invade newer areas namely Bangladesh, Myanmar, Sri Lanka, Thailand (FAO, 2019b), and Southern China (Yunnan Province) in early January 2019 (Wu et al., 2019); Malaysia in March 2019; Indonesia and Hong Kong in April 2019; Taiwan in May/June 2019; Laos and Vietnam in April 2019 (USDA, 2019); Philippines (FAO, 2019c), South Korea, and Japan in June 2019; and Australia by 2020 (FAO, 2020). FAW is a polyphagous insect pest and feeds on 353 host plants belonging to 76 families (Sparks, 1979; Capinera, 2017; Montezano et al., 2018; Padhee and Prasanna, 2019) with a preference for graminaceous plants (Malo and Hore, 2020). Among various host plants, maize is the most preferred host for FAW. The availability of maize in a large area round the year in different stages coupled with the high reproductive capacity of FAW has led to the increased incidence of FAW in maize [USAID (United States Agency for International Development), 2018]. FAW is reported to cause yield losses of 20–50% in maize (Early et al., 2018; Banson et al., 2019).

The management of FAW is critical to avoid losses caused due to its infestation. FAW being an invasive insect pest is relatively new to the maize-based crop ecosystem in Asia and elsewhere, and there was little information available on the management of FAW through different ways and means (Prasanna et al., 2021). Based on the unique mode of action, the chemical insecticides, namely, anthranilic diamides, avermectins, and spinosyns, have been registered in India for immediate management of FAW. However, long-term use of chemicals to control FAW can lead to the development of insecticide resistance, resurgence, toxic effects on non-target organisms, and environmental pollution (Togola et al., 2018). Further, the effectiveness of the above insecticides on mature FAW larvae is limited as the larva feed inside the whorls (cryptic feeding behavior) (De Groote et al., 2020). Moreover, the newer generation insecticides as seed treatment and foliar sprays are punitive (Midega et al., 2012) for smallholder maize farmers in India. In addition, there are other options like the use of entomopathogens to control FAW as they are natural regulators of insect pest populations. However, their performance is less due to shorter shelf life, slow working mode, higher production cost, and also require specific environmental conditions, such as higher humidity (Jaronski, 2010). Hence, a viable option is to rely on host plant resistance (HPR), which is economically viable and environmentally sustainable. HPR is also easy to use and can be well-integrated into multi-tactic IPM programs (Mortensen, 2013).

Identification of resistance sources followed by their utilization in the regular breeding program to develop and deploy resistant cultivars can be effective not only to reduce the losses caused by insect pests but also to keep pest populations below an economic threshold level. Even though FAW attacks all stages of the maize crop, the most sensitive one is the seedling stage. Thus, it is the most important and critical to identify the resistant sources at the seedling stage. Identification of resistant sources especially at an early stage will substantially aid in breeding for resistance to FAW. Further, understanding the basis of resistance will help in developing breeding schemes to accelerate the development of insect resistance commercial cultivars (Russell, 2013). The nature and degree of damage caused by FAW on maize vary depending upon larval instars, growth stage of the crop, and resistance/susceptibility reaction of a particular genotype against FAW damage. In addition, environmental conditions also play a role, for example, early to mid-instar larvae are washed out under continuous and heavy rains thus reducing the damage by FAW.

Until recently (2018), FAW was not found in India, thus there were no reports from India on screening techniques under artificial infested conditions and damage ratings to date. Therefore, information on the availability of sources of resistance among maize hybrids and parental lines against FAW was not available. Under Indian climatic conditions, there is an utmost need to optimize the screening technique against FAW with a damage rating scale. Accordingly, the present study was conducted with the following objectives: (i) Optimisation of the screening technique by comparing injury levels among maize genotypes and (ii) Identification of potential sources of resistance to FAW under artificial infestation conditions based on the optimized screening technique. The results of the present study will serve as the template for future studies.

## Materials and Methods

### Rearing of FAW Culture

The neonate larvae of FAW were collected from infested plants in the field at the Winter Nursery Centre (WNC), Hyderabad, India (17.325429″N latitude and 78.397010″E longitude), as the initial culture or population. Larvae were reared in the laboratory at 28 ± 1°C, 65 ± 10% RH under 16 h light/8 h dark photoperiod initially for 5 days in a group of 50–100 in a plastic jar containing 2–3 mm layer of chickpea flour–casein-based artificial diet at the bottom side of the jar (Singh and Rembold, 1992). Later, the larvae were transferred individually to 12-well plates (HiMedia) with each cell 2.5 cm in diameter and 2.3 cm in depth to avoid cannibalism and maintained until pupation. The pupae were sterilized with 2% sodium hypochlorite solution and kept in groups of 25–50 in plastic jars containing soil. After adult emergence, 10 pairs of FAW moths were released inside an oviposition cage of 30 × 23 cm (length × diameter) dimension. The adult moths were provided with a 10% honey solution in a cotton swab for feeding and the blotting paper strips were hung inside the cage as an oviposition substrate. The blotting paper strips were replaced daily and the eggs collected on the paper were sterilized with a 10% formalin solution. Later, the eggs were allowed to hatch and the larvae were transferred with a hairbrush to plastic jars containing an artificial diet (Singh and Rembold, 1992). The lab-grown neonate larvae were used for an artificial infestation to standardize the screening technique.

### Optimization of Screening Technique in Net House

The screening technique was standardized by selecting six genotypes comprising four inbred lines, namely BML 6, BML 7, CM 500, CM 400, and two hybrids namely DHM 117 and COHM 8. Each of the six genotypes was grown in four replications separately for each of the different larval loads at two phenological stages namely V_5_ (collar of 5th leaf visible) and V_7_ (collar of 7th leaf visible). The number of neonate larvae released consisted of 5, 10, 15, and 20 per plant in each of the six genotypes. The genotypes were raised in a 2-m row by providing optimum growing conditions; the spacing between rows and plant-to-plant distance followed was 75 and 20 cm, respectively. Initially, the sowing was taken up manually on ridges at a depth of 5 cm below the soil surface by putting two seeds per hill but finally, after germination, one plant per hill was maintained by thinning out excess plants. The experiment was conducted separately for each phenological stage (V_5_ and V_7_) on black loamy soil in the insecticide-free net house at WNC, Hyderabad, India, during July to October 2020.

The neonate larvae were released during morning hours (9.00–10.00 a.m.) using a fine camel hair brush separately for each stage. The observations were recorded visually on the degree of leaf-feeding damage on the 7th, 14th, and 21st day after infestation (DAI) for each larval dose separately for each plant stage and compared injury levels among maize genotypes. The leaf damage rating (LDR) was given as per a precisely developed scale of 1 (healthy plant) to 9 (complete whorl damaged); modified from Williams et al. (1989), Davis and Williams (1992), Ni et al. (2011), Prasanna et al. (2018), and Toepfer et al. (2021). The observations were recorded on the type of holes, the number of scraped leaves, and the number and the length of lesions on each of 50 plants for each rating (1-9).

### Evaluation of Maize Genotypes for FAW Resistance in Net House

The experimental material consisted of 38 diverse maize genotypes which included 11 and 26 inbred lines developed at ICAR-IIMR and CIMMYT, respectively, and one wild ancestor, *Zea mays* ssp. *parviglumis* (Accession No-Ames 21797; Pedigree I.A.16; Location: National Bureau of Plant Genetic Resources, New Delhi). The genotypes were comprised of different kernel colors and textures and derived from diverse genetic background (Table 1). The genotypes were screened under artificial infestation based on the optimized screening technique, that is, by comparing injury levels caused by FAW. Twenty neonate larvae/plants were released at the V_5_ stage in an insecticide-free net-house during 2020–2021. The experiment was laid out in randomized complete block design in three replications of 2 m row for assessing resistance/susceptibility reaction based on LDR using a 1–9 scale.

**Table 1:** Maize genotypes used in the study.

**S. No**.	**Genotype**	**Category**	**Grain color/texture**	**Source germplasm**
Ist Experiment				
1	BML 6	Late	Yellow, Semi Dent	SRRL65-B96-1-1-2-#-2-2-1-X-1-1
2	BML 7	Medium	Orange, Flint	[×2Y Pool × CML 226]-B 98 R-1-1-1-⊗ b -⊗ b -⊗ b -⊗ b -⊗ b -⊗ b
3	CM 500	Early	Yellow, Flint	Antigua-Gr-I
4	CM 400	Late	White, Dent	Tennessee-29
5	DHM 117	Medium	Yellow, Flint	BML6/BML7
6	COH(M) 8	Medium	Yellow, Semi Dent	UMI 1201 × UMI 1230
IInd Experiment				
1	DMRSC-2	Medium	Yellow, Flint	WNC2011R-16069
2	DML163-1	Medium	Yellow, Flint	MRCH6340-6-3-1-5-B-B
3	DQL780-2	Medium	Yellow, Flint	Derived from HQPM 5
4	IML12-215	Medium	Yellow, Flint	Derived from BML6 × DML 177-1-7-6
5	DMRE 63	Early	Orange, Flint	WNZPBTL 9
6	E 57	Medium	Orange, Flint	WNZPBTL 6
7	AEBY-1	Early	Orange, Flint	IIMPRSBT POOL
8	NAI-178A	Late	Yellow, Semi Dent	Pop 146C5MH134-#-2-1
9	ENT 2-3	Medium	Light Orange, Flint	WNZPBTL 1
10	PFSR-R3	Medium	Yellow, Flint	JCY3-7-1-2-3-b-1-1-2-3-1-1-1-1
11	MIL 1-11	Late	Yellow, Flint	((G32-F32/P42-F258)-11-1-2-B)/(*Zeadiploperennis*)- CM111'*Zeadiploperennis*'CM111
12	CML 59	Medium	Yellow, Semi Flint	ANT11305-1-1-B11-2-3-B
13	CML 60	Medium	Yellow, Flint	ANT11305-1-1-B11-2-3-B
14	CML 67	Late	Semi Dent	(ANTGP2-5-#-1/ANT38586-1)-6-B-4-2-2-5-B
15	CML 70	Late	Flint	(ANTGP2-5-#-1/ANT38586-1)-6-B-2-3-1-B
16	CML 71	Late	Flint	ANTGP2-5-#-1-2-1-1-5-5-7-B
17	CML 72	Late	Flint	ANTGP2-5-#-1-2-1-1-3-3-1-B
18	CML 73	Late	Flint	ANTGP2-5-#-1-2-3-B-1-1-1-B
19	CML 121	Medium	Yellow, Dent	(PI218191/PI209135//PI226685/P1317328-3/P47/MPSWCB4)-6-3-1-5-1-B/(MP704/MP78-518)-8-3-4-B-1-4-2-3-B
20	CML 122	Medium	Yellow, Dent	(MP704/MP78-518)-8-3-4-B-4-2-3-B
21	CML 139	Late	Yellow, Semi Flint	MP78-518-15-B
22	CML 141	Late	White, Flint	P62-C5-FS24-5-3-2-1-B-B-2-B
23	CML 144	Late	White, Flint	P62-C5-FS182-2-1-2-B-B-3-1-B
24	CML 202	Late	White, Semi Dent	ZSR923-B*4-5-1-B
25	CML 330	Early	White, Semi Dent	(SUWAN8422)/(P47/MP78-518)-#-7-1-1-1-1-1-B
26	CML 331	Early	White, Semi Dent	(SUWAN8422)/(P47/MP78-518)-#-183-1-2-1-2-2-B
27	CML 332	Early	White, Semi Dent	(SUWAN8422)/(P47/MP78-518)-#-183-1-7-3-1-2-B
28	CML 334	Early	White, Semi Dent	P590-C3-F374-2-1-2-B-#-3-3-B
29	CML 335	Early	Yellow, Semi Flint	(SEYF)/(P47/MP78-518)-B-23-1-5-1-1-2-B
30	CML 336	Early	Yellow, Dent	(TL8645)/(P47/MP78-518)-B-24-1-1-4-1-3-B
31	CML 337	Early	Yellow, Dent	(TL8645)/(P47/MP78-518)-B-24-1-3-2-1-2-B
32	CML 338	Early	Yellow, Semi Flint	P590B-F84-3-3-5-3-1-1-B
33	CML 345	Late	White, Flint	P390SCB-C1-F72-1-1-1-1-#-6-B
34	CML 346	Late	White, Flint	AC90390SCBSR-F430-1-1-2-3-2-2-B
35	CML 405	Late	White, Flint	LAPOSTASEQ-C0-B*3-12-1-1-B
36	CML 452	Medium	Yellow, Semi Dent	AC8328BN-C6-166-1-1-1-B
37	CML 501	Early	Yellow, Semi Flint	(CL02709/V)-B*3-1-1-B
38	*Zea mays parviglumis*	Late	Brown, conical	Wild ancestor, Accession No-Ames 21797; Pedigree I.A.16; Location: National Bureau of Plant Genetic Resources, New Delhi

### Statistical Analysis

The analysis related to optimization of screening technique was carried out using SAS 9.4, Mixed Procedure. Repeated Measurement ANOVA was carried out with genotypes, neonate count as fixed between-subject factor, days after infestation was considered as a fixed within-subject factor, and replication was considered as a random factor. This model was tried separately for each phenological stage. Different variance-covariance structures were tried for the within-subject factor and using AIC (Akaike's information criterion), AICC (Corrected Akaike's information criterion), BIC (Sawa Bayesian information criterion) criteria, the best structure was identified. The unstructured variance-covariance structure was found to have the least in all Fit Statistics and considered for estimating the Least square means in both phenological stages. The LDR data pertaining to the screening of maize germplasm was subjected to RBD analysis using the SAS version 9.3. The mean values were separated using LSD at *P* < 0.05 (SAS Institute, 2011).

## Results

### Rating Scale With a Detailed Description of Foliage Damage Symptoms

**Figure 1:**
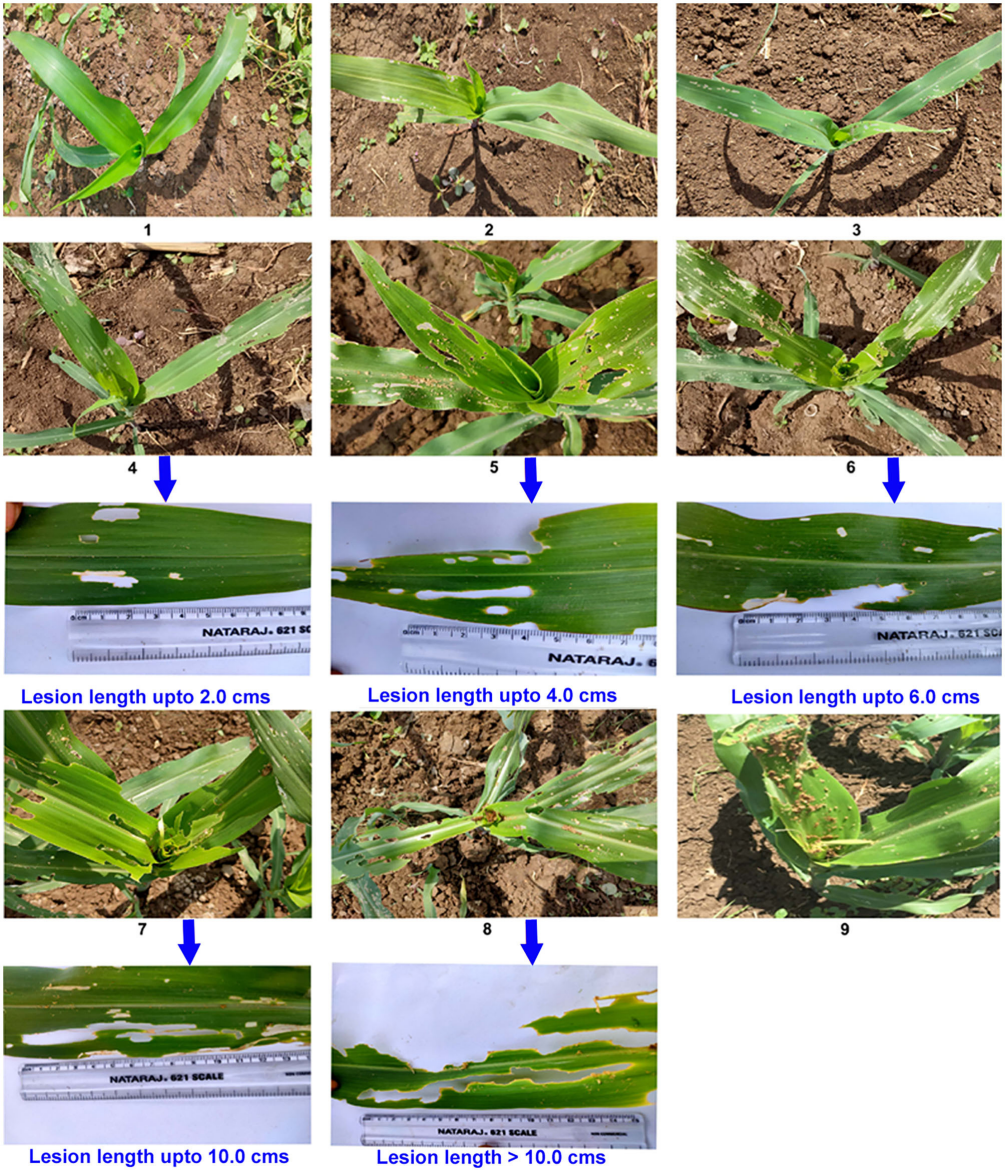
Simple 1–9 whole plant leaf damage scale for the fall armyworm.

The modified LDR of a 1–9 scale was developed with a detailed description of leaf damage based on the observed symptoms (Table 2, Figure 1). The LDR scale presented here was the modification in the description of leaf damage symptoms earlier given by Williams et al. (1989), Davis and Williams (1992), Ni et al. (2011), Prasanna et al. (2018), and Toepfer et al. (2021), and it was used in the characterization of maize germplasm into resistant (1–4), moderately resistant (>4–6), and susceptible (>6–9).

**Table 2:** Leaf Damage Rating (LDR) scale to categorize maize germplasm for resistance to FAW (Modified from Davis and Williams, 1992; Ni et al., 2011).

**Rating**	**Description/Symptoms**
1	Healthy plant/No damage/Visible symptoms
2	Few short/pin size holes/scraping on few leaves (1–2)
3	Short/pin size holes/scraping on several leaves (3–4)
4	Short/pin size holes/scraping on several leaves (5–6) and a few long elongated lesions (1–3 Nos) up to 2.0 cm length present on whorl and or adjacent fully opened leaves
5	Several holes with elongated lesions (4–5 Nos) up to 4.0 cm length and uniform/irregular shaped holes present on whorl and or adjacent fully opened leaves
6	Several leaves with elongated lesions (6–7 Nos) up to 6.0 cm length and uniform/irregular shaped holes present on whorl and adjacent fully opened leaves
7	Several long lesions (>7 Nos) up to 10 cm length and uniform/irregular shaped holes common on one-half of the leaves present on whorl and adjacent fully opened leaves
8	Several long lesions >10 cm length and uniform/irregular shaped holes common on one half to two-thirds of leaves present on whorl and adjacent fully opened leaves
9	Complete defoliation of whorl of the plant

### Response of Maize Genotypes to FAW at V_5_ and V_7_ Phenological Stages

In the present study, the response of maize genotypes was studied by comparing injury levels caused by FAW neonates. The results of three-way ANOVA showed that genotype, neonate counts, days after infestation, and interaction effects, *viz*., genotype × days after infestation, neonate counts × days after infestation, and genotype × neonate counts × days after infestation, significantly influenced the LDR score at 7, 14, and 21 DAI (Table 3) at V_5_ phenological stage. Genotype, neonate counts, and days after infestation significantly influenced the LDR scores at the V_7_ stage also. The interaction between these factors also had significant effects on LDR with the exception of neonate counts × days after infestation.

**Table 3:** ANOVA for the Least Square means of three-way interaction (Genotype, Neonate counts, and Days after infestation) in net house conditions.

**Effect**	**Numerator DF**	**Denominator DF**	***F*** **value**	**Pr > *F***
V_5_ Phenological stage				
Genotype	5	69.6	65.02	<0.0001
Neonate counts	3	69.6	528.69	<0.0001
Days after infestation	2	72	128.46	<0.0001
Genotype ^*^ Days after infestation	10	72	18.51	<0.0001
Neonate count ^*^ Days after infestation	6	72	6.95	<0.0001
Genotype ^*^ Neonate counts ^*^ Days after infestation	45	69.8	5.23	<0.0001
V_7_ Phenological stage				
Genotype	5	68.1	60.71	<0.0001
Neonate counts	3	68.1	242.79	<0.0001
Days after infestation	2	72	110.70	<0.0001
Genotype ^*^ Days after infestation	10	72	21.15	<0.0001
Neonate counts ^*^ Days after infestation	6	72	0.98	0.4458
Genotype ^*^ Neonate counts ^*^ Days after infestation	45	70.5	4.90	<0.0001

**Figure 2:**
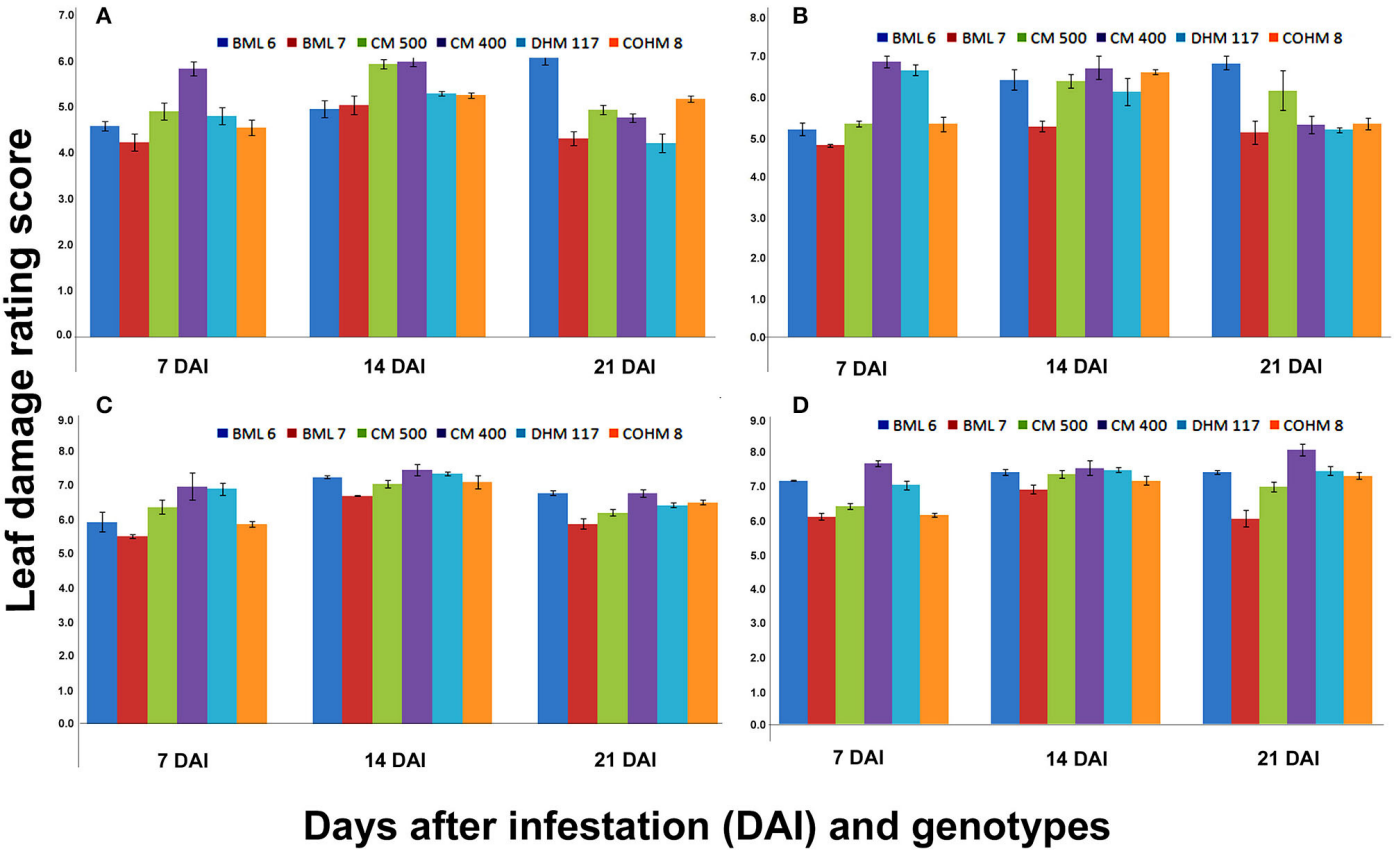
Leaf damage rating (LDR) scores at V_5_ phenological stage of maize under artificial infestation in net house conditions at 7,14 and 21 DAI.

**Figure 3:**
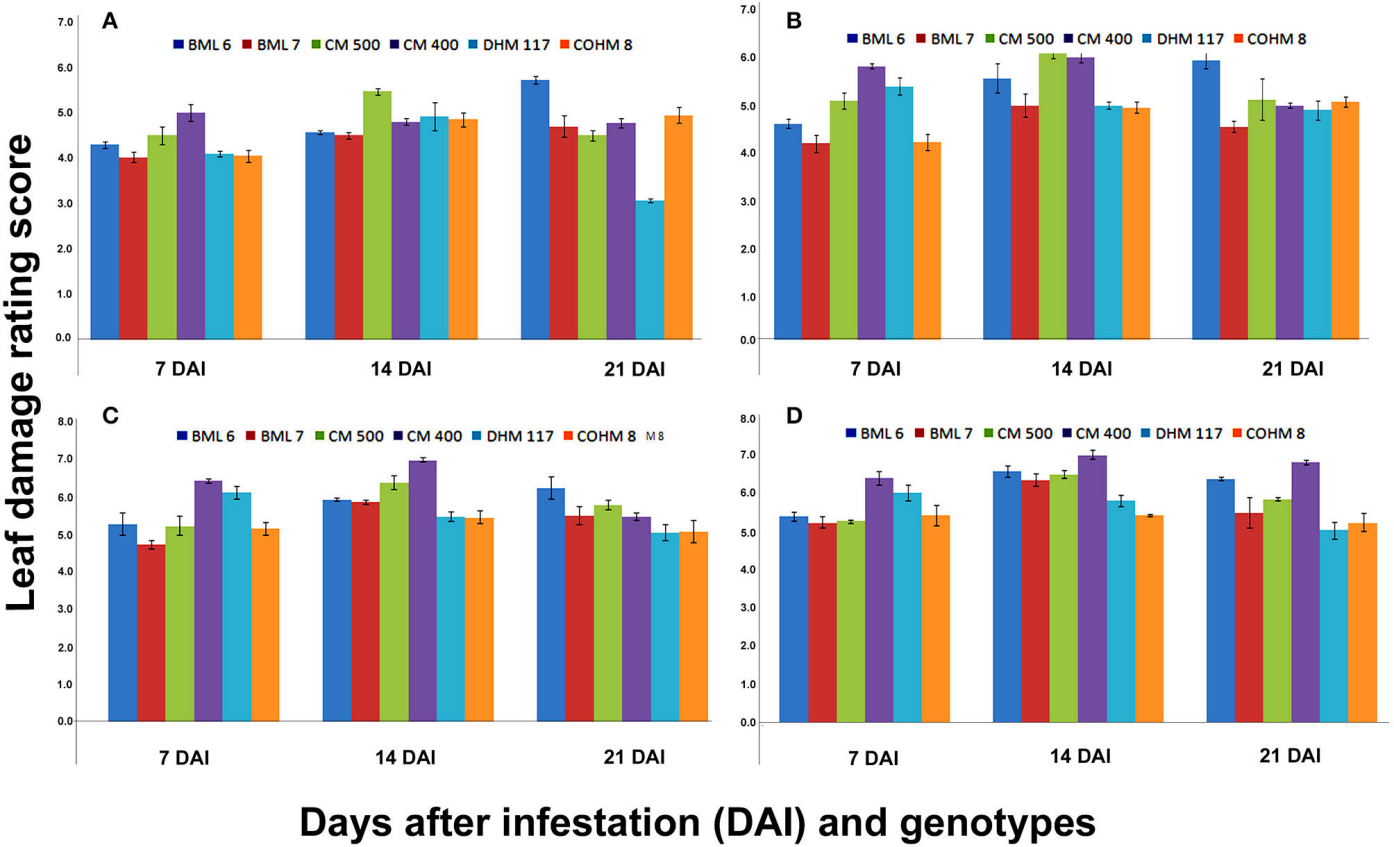
Leaf damage rating (LDR) scores at V_7_ phenological stage of maize under artificial infestation in net house conditions at 7, 14 and 21 DAI.

The results of three-way ANOVA indicated significant differences in LDR score among maize genotypes at 7, 14, and 21 DAI when different counts of larvae were released at V_5_ (Figure 2) and V_7_ phenological stages (Figure 3). The V_5_ stage plants when challenged with five larvae/plant, and the genotypes showed significant differences for LDR at 7 DAI that varied from 4.23 (BML 7) to 5.83 (CM 400), while it was in the range of 4.8 (BML 7) to 6.88 (CM 400), 5.53 (BML 7) to 7.00 (CM 400), and 6.13 (BML 7) to 7.68 (CM 400) when the plants were challenged with 10, 15, and 20 larvae per plant, respectively. Similarly, the LDR observed at 14 DAI ranged from 4.95 (BML 6) to 5.98 (CM 400), 5.28 (BML 7) to 6.73 (CM 400), 7.08 (CM 500) to 7.48 (CM 400), and 6.93 (BML 7) to 7.55 (CM 400) when challenged with 5, 10, 15, and 20 larvae/plant, respectively. The LDR score results observed at 21 DAI ranged from 4.21 (DHM 117) to 6.18 (BML 6), 5.13 (BML 7) to 6.85 (BML 6), 5.9 (BML 7) to 6.81 (CM 400, BML 6), and 6.08 (BML 7) to 8.08 (CM 400) for 5, 10, 15, and 20 larvae per plant, respectively (Figure 2).

The LDR score recorded on V_7_ phenological stage plants after 7, 14, and 21 DAI with different counts of neonate larvae (5, 10, 15, and 20) also showed the significant differences among maize genotypes (Figure 3). The LDR recorded at 7 DAI varied from 4.02 (BML 7) to 5.0 (CM 400), 4.2 (BML 7) to 5.82 (CM 400), 4.72 (BML 7) to 6.42 (CM 400), and 5.27 (BML 7) to 6.42 (CM 400) when exposed to 5, 10, 15, and 20 larvae/plant, respectively. Similarly, a significant difference among the genotypes in LDR was observed at 14 DAI wherein the LDR ranged from 4.5 (BML 7) to 5.47 (CM 500), 4.95 (COHM 8) to 6.12 (CM 500), 5.45 (COHM 8) to 6.97 (CM 400), and 5.45 (COHM 8) to 7.02 (CM 400) when challenged with 5, 10, 15, and 20 larvae/plant, respectively. Similar results were also obtained at 21 DAI, and significant differences in LDR were observed among the genotypes. The LDR ranged from 3.07 (DHM 117) to 5.72 (BML 6), 4.9 (DHM 117) to 5.95 (BML 6), 5.05 (COHM 8, DHM 117) to 6.22 (BML 6), and 5.07 (DHM117) to 6.82 (CM 400) when 5, 10, 15, and 20 larvae/plant were released, respectively. The results obtained in the present study indicate that the LDR score depends on genotype, the counts of larval released, and days after infestation.

### Evaluation of Maize Genotypes Grown Under Net House Conditions for Resistance to FAW

Significant differences in the LDR scores among the genotypes against FAW damage were observed by comparing injury levels caused by FAW neonates which ranged from 3.29 (CML 73) at 7 DAI to 8.50 (E 57) at 21 DAI (Tables 4, 5). Out of the 38 genotypes screened against FAW in the net-house under artificial infestation, 19 genotypes recorded an LDR score of <4 at 7 DAI, while 13 genotypes recorded LDR between 4.1 and 6.0, and the remaining six genotypes exhibited LDR>6. At 14 DAI, seven genotypes exhibited significantly lower LDR scores (<4) and six genotypes showed higher LDR (>6.0), whereas, in the rest of the genotypes (25), a medium LDR score was observed. At 21 DAI, 15 genotypes recorded LDR scores in the range of 2.50–3.99 with the lowest score in *Z. mays* ssp. *parviglumis* (2.50), while LDR score >4.0–6.0 was observed in 18 genotypes; the remaining five genotypes exhibited LDR ranging between 8.25 and 8.50. The minimum overall mean LDR (<4.0) was observed in eight genotypes namely DMRE 63 (3.52), DML 163-1 (3.74), CML 141 (3.85), MIL 1-11(3.86), CML 71 (3.93), CML 337 (3.96), and CML 345 (3.99) including wild ancestor, *Z. mays* ssp. *parviglumis* (3.25). The remaining CML series exhibited LDR between 4.02 and 4.94 and other genotypes, such as DMRSC-2, E 57, AEBY-1, NAI-178A, ENT 2-3, and PFSR-R3, showed LDR in the range of 6.64–8.20.

**Table 4:** ANOVA for the leaf damage rating of maize germplasm on different days after infestation (DAI) in net house conditions.

**Source**	**DF**	***F*** **value**	**Pr (>*F*)**
7 DAI			
REP	2	4.87	0.0103
TRT	37	22.20	<0.0001
Error	74		
Corrected Total	113		
14 DAI			
REP	2	1.46	0.2381
TRT	37	10.56	<0.0001
Error	74		
Corrected Total	113		
21 DAI			
REP	2	2.29	0.1087
TRT	37	11.81	<0.0001
Error	74		
Corrected Total	113		

**Table 5:** Leaf damage rating of maize germplasm on different days after infestation (DAI) in net house conditions.

**S. No**.	**Genotypes**	**7 DAI**	**14 DAI**	**21 DAI**	**Over all mean**	**Response**
1	*Z. mays parviglumis*	3.55^jkl^	3.69^ij^	2.50^j^	3.25	R
2	DMR E63	3.46^kl^	3.70^hij^	3.41^fghi^	3.52	R
3	DML163-1	4.25^fghij^	3.71^hij^	3.25^ijh^	3.74	R
4	DQL780-2	4.30^fghi^	4.7^cdefghi^	3.66^efghi^	4.22	MR
5	IML12-215	4.79^ef^	4.07^efghij^	3.96^defghi^	4.27	MR
6	MIL 1-11	3.97^ghijkl^	4.49^cdefghij^	3.11^ij^	3.86	MR
7	CML 59	3.95^ghijkl^	5.46^c^	4.85^bcde^	4.75	MR
8	CML 60	3.69^ijkl^	4.42^cdefghij^	4.66^bcdefg^	4.26	MR
9	CML 67	4.20^fghijkl^	4.99^cdefg^	4.01^cdefghi^	4.40	MR
10	CML 70	3.44^l^	5.26^cde^	5.98^b^	4.89	MR
11	CML 71	3.60^ijkl^	3.69^ij^	4.39^cdefghi^	3.93	R
12	CML 72	3.32^l^	4.81^cdefghij^	4.54^cdefgh^	4.22	MR
13	CML 73	3.29^l^	4.76^cdefghij^	4.74^bcdef^	4.26	MR
14	CML 121	3.90^hijkl^	4.60^cdefghij^	4.08^cdefghi^	4.19	MR
15	CML 122	3.61^ijkl^	4.58^cdefghij^	3.99^defghi^	4.06	MR
16	CML 139	3.38^l^	5.13^cdef^	3.97^defghi^	4.16	MR
17	CML 141	4.67^efg^	3.76^ghij^	3.12^ij^	3.85	R
18	CML 144	3.76^hijkl^	5.37^cd^	4.77^bcdef^	4.63	MR
19	CML 202	3.66^ijkl^	4.24^cdefghij^	4.16^cdefghi^	4.02	MR
20	CML 330	3.66^ijkl^	4.79^cdefghij^	5.36^bc^	4.60	MR
21	CML 331	3.40^kl^	5.15^cdef^	4.41^cdefghi^	4.32	MR
22	CML 332	3.75^hijkl^	4.42^cdefghij^	5.17^bcd^	4.45	MR
23	CML 334	4.31^fghi^	5.24^cdef^	5.16^bcd^	4.90	MR
24	CML 335	5.30^cde^	5.45^c^	4.08^cdefghi^	4.94	MR
25	CML 336	3.90^hijkl^	4.14^defghij^	4.37^cdefghi^	4.14	MR
26	CML 337	4.33^fghi^	3.59^j^	3.96^defghi^	3.96	R
27	CML 338	4.49^fgh^	4.88^cdefghij^	3.30^ghij^	4.22	MR
28	CML 345	4.33^fghi^	4.43^cdefghij^	3.2^ijh^	3.99	MR
29	CML 346	5.55^cd^	4.00^efghij^	3.85^defghi^	4.47	R
30	CML 405	4.90^def^	4.95^cdefgh^	3.27^jhi^	4.37	MR
31	CML 452	4.65^efg^	4.46^cdefghij^	3.16^ij^	4.09	MR
32	CML 501	4.00^ghijkl^	4.77^cdefghij^	4.26^cdefghij^	4.34	MR
33	DMRSC-2	6.96^ab^	7.04^b^	5.92^b^	6.64	S
34	ENT 2-3	6.10^c^	8.45^a^	8.43^a^	7.66	S
35	E57	6.91^b^	8.39^a^	8.50^a^	7.93	S
36	NAI-178A	7.34^ab^	8.48^a^	8.25^a^	8.02	S
37	AEBY-1	7.29^ab^	8.43^a^	8.46^a^	8.06	S
38	PFSR-R3	7.69^a^	8.47^a^	8.44^a^	8.20	S
	F	22.20	10.56	11.81		
	P	<0.0001	<0.0001	<0.0001		
	LSD	0.74	1.25	1.36		

*Means with the same letter are not significantly different. Each value represents the mean of three replications*.

*R, Resistant; MR, Moderately Resistant; S, Susceptible*.

## Discussion

The invasive insect pest of maize, FAW, with a serious threat to sustained maize production in the country, demands immediate and urgent action to address the challenge in both the short- and long-term. In this context, the identification of sources of resistance to invasive insect pests is one of the economically viable, environmentally friendly, and sustainable long-term measures. Subsequently, the sources of resistance could serve as valuable genetic resources for resistance breeding. The integration of host plant resistance could also be exploited by integrating into the IPM. However, to identify the resistance source, a reliable screening technique against FAW under artificial infestation is the most important as evaluation of maize germplasm to identify resistance sources under natural infestation is not reliable. More so because the insect pest population and the damage caused by FAW vary across locations and seasons. Thus, it demands the optimization of screening techniques with an appropriate rating scale under artificial infestation for the effective identification of insect resistance sources.

### Rating Scale With a Detailed Description of Foliage Damage Symptoms

In the earlier developed rating scales, the leaf damage severity is visually assessed based on whorl and furl damage (0-9 scale) (Davis and Williams, 1992); all leaves of the plant (0–9 scale) (Williams et al., 1998). Furthermore, in the rating scale mentioned by earlier researchers, symptoms based on the number of lesions and their length have not been described in quantifiable terms at 5, 6, 7, and 8 ratings (Williams et al., 1989); 5, 7, and 8 ratings (Davis and Williams, 1992; Ni et al., 2011); 6, 7, and 8 ratings (Prasanna et al., 2018); 7 and 8 ratings (Toepfer et al., 2021). Moreover, there will be an observer bias during the rating process related to the descriptive part of the score in the earlier ones (Tversky and Kahneman, 1974). In the present study, the descriptive part of each damage rating (1–9 scale) was modified based on the visual assessment of foliage damage on the whole plant. The description of lesion length in this article particularly at 6, 7, and 8 ratings is precise and provides information about the degree of susceptibility of the tested genotype. The scores between 6, 7, and 8 were described based on the length of elongated lesions as up to 6.0, 10.0, and >10 cm, respectively. This rating scale describes the proportion of damaged leaves and elongated lesions so that fine differences in damage levels can be estimated. It is noteworthy to mention that the present modified rating scale (1–9) is easily understandable, the response of genotypes can be easily characterized, it is not labor intensive, and consistent for large-scale trials. This rating scale can be used for 7-, 14-, and 21-day assessments after artificial infestation with FAW neonates. However, the modified scale is suitable only for the vegetative growth stage of the plant. The simple characterization of genotypes as resistant, moderately resistant, and susceptible is sufficient enough to separate resistant genotypes from susceptible which is the prime objective of insect pest screening.

### Response of Maize Genotypes to FAW at V_5_ and V_7_ Phenological Stages in Net House

The screening technique under artificial infestation should be as near-natural as possible. This delicate balance needs to be maintained while screening under artificial infestation that the insect infestation pressure should be high enough which separates susceptible from resistant material, and at the same time, it must not be so high that the lines with moderate resistance are lost due to difficulty to distinguish from highly susceptible lines because of very high insect load. Earlier several studies have been conducted for evaluation of maize germplasm resistant to FAW under artificial infestation with different doses of FAW neonates, that is, two applications of 15 neonate larvae at V_8_ or V_12_ stage (Wiseman, 1989); 30 neonates at V_4_ or V_8_ stage (Davis et al., 1996); 15 neonate larvae at V_6_-V_8_ stage (Alvarez and Filho, 2002); 15–20 neonate larvae at V6 stage (Ni et al., 2011); 5–10 neonate larvae or 20 black-head stage eggs (Prasanna et al., 2018, 2021) at V5 stage; and 30 neonates at V_7_ stage (Womack et al., 2020). In the present study, the response of maize genotypes was studied by feeding on 5, 10, 15, and 20 neonate larvae at the V_5_ and V_7_ phenological stages on six different maize genotypes to optimize the screening technique. The genotypes CM 400 and BML 6 consistently suffered the most injury, while BML 7 and COHM 8 suffered a moderate injury at the V_5_ and V_7_ phenological stages. The results did not show much variation among the genotypes in terms of LDR when fed on 5 and 10 neonate larvae as compared to 15 and 20. As expected, LDR increased with the larval load in both phenological stages, since increasing the number of herbivores is likely going to increase the damage they inflict. Another important parameter that has affected the LDR is days after infestation with FAW neonate larvae. In general, it was observed that the LDR was higher at 14 DAI compared to 7 DAI and then further decreased at 21 DAI at both V_5_ and V_7_ phenological stages. The important understanding from the study emerged was that the rating at 7 DAI is premature since larvae are in the mid-growth stage (first to third instar) have not attained their peak damage level. This is because the first to third instar larvae of FAW are quite small and eat 2% of the total foliage consumed in its life cycle, while the 4th, 5th, and 6th instars larvae eat 4.7, 16.3, and 77.2% and heavily defoliate the crop (Sparks, 1979). As the larvae undergo pupation when the rating is taken at 21 DAI, the plants do not reflect the actual damage caused due to larvae as host plants start to recover from damage. Therefore, the rating recorded at 14 DAI provides actual host plant and insect pest interaction reflecting the complete leaf damage caused due to FAW and also the response of the host plant against the FAW infestation. Moreover, the feeding rate of earlier instars (First and second) is very less (Ren et al., 2020) and this might be the reason for the low rating of 7 DAI. The released neonate larvae become fourth to fifth instar feed heavily by the time when data are taken at 14 DAI resulting in higher damage. In the present study, the genotype BML 6 consistently showed susceptible reaction even at 21 DAI when different doses of FAW neonates were released.

In general, the larval infestation across genotypes caused significantly higher LDR scores at V_5_ stage plants than in V_7_ stage at 7, 14, and 21 DAI. This might be due to the increased tolerance level of V_7_ stage plants as compared to V_5_ stage plants to FAW damage. The present result is in agreement with the finding of Wiseman and Isenhour (1993) who reported that maize genotypes at 12 leaf stage tolerated FAW damage compared to plants at 8 leaf stage. The level of damage due to FAW infestation at V_5_ stage across genotypes was substantial when 20 neonate larvae/plant were released as compared to 5, 10, and 15 neonates. The present study confirms that the LDR score is the result of the interaction effect of the host plant or genotype, neonate load, and days after infestation. Since the V_5_ stage plants across genotypes showed significantly higher LDR scores with 20 neonate counts, the best evaluation stage to screen maize germplasm is the V_5_ phenological stage by infesting with at least 20 neonate larvae/plant in an insecticide-free net house.

Another important parameter to be considered while optimizing screening methodology is the cannibalism behavior of FAW. However, the frequency of cannibalism depends upon the larval stage, larval density, plant characteristics (Hodek and Honek, 1996), availability of food, and so on. In general, the older larvae of FAW (fifth and sixth instar) exhibit this behavior on younger instars compared to same-age larvae (Chapman et al., 1999, 2000). In field conditions, it is observed that early instars feed in the same whorl whereas mature larvae never cohabit as explained by Carvalho and Silveira (1971). Cannibalism also depends upon the degree of crowding which is greater only at higher larval densities (Chapman et al., 1999). Therefore, in the present optimized screening technique, as same-age neonate larvae were released at a single time with optimum larval load under net house conditions, the effect of cannibalism is minimal.

### Evaluation of Maize Genotypes Grown Under Net House Conditions for Resistance to FAW

The second part of the present study validates the result obtained in the first experiment. A diverse set of maize germplasm (38) was screened to compare the injury levels among different genotypes against FAW infestation and to identify sources of resistance to FAW. Based on the results obtained in the standardized screening experiment, 20 FAW neonate larvae were released on each genotype at the V_5_ phenological stage. The response of different genotypes against FAW was recorded as LDR scores at 7, 14, and 21 DAI. The observations recorded in the second experiment validated the observations made in the standardization of screening technique as the LDR score in most of the genotypes was low (Average LDR-4.43) due to FAW infestation at 7 DAI, while it was increased at 14 DAI (Average LDR-5.26). However, the FAW damage decreased at 21 DAI (Average LDR-4.70) in most of the genotypes. The results of the validation are in perfect conformity with the standardization experiment. Thus, the results further corroborated the utility of the modified screening technique developed in the present study. Further, significant variation in LDR was observed among maize germplasm at 14 DAI as compared to 7 and 21 DAI. The present result, therefore, indicates that infestation of maize genotypes with 20 neonates/plants at an early stage (V_5_) and considering FAW damage at 14 DAI is the most appropriate to characterize maize germplasm. Similar observations were also made by Buntin (1986) who reported that FAW infestation at the early whorl stage causes more damage than late whorl stages. Based on the response of maize genotypes to FAW damage, seven lines namely DMRE 63, DML163-1, CML 71, CML 141, CML 337, CML 346, and wild ancestor *Z. mays* ssp. *Parviglumis* were classified as potentially resistant (LDR 1–4 at 14 DAI), while 25 were potentially moderately resistant (LDR 4.1–6 at 14 DAI) and the remaining six genotypes (LDR 6.1–9 at 14 DAI) were seemingly susceptible (Table 5). The present study identified seven genotypes potentially useful for FAW resistance breeding.

Previously several native resistant sources for FAW have been identified in different germplasm. Wiseman et al. (1967) reported resistant sources in Caribbean maize germplasm namely Cuba Honduras 46-J, Eto Amarillo. Subsequently, several other sources of resistance were reported namely GT-CEW-RS8, GT-RI4 (Widstrom et al., 1975, 1984), Tuxpeno germplasm (Smith, 1982), and Antigua 2D × (B10 × B14) (Wiseman et al., 1996). In addition, USDA-Mississippi temperate maize inbreds, *viz.*, Mp 496; Mp701-Mp708, Mp713; Mp714; Mp716 (Williams and Davis, 1980, 1982, 1984, 2000, 2002; Scott and Davis, 1981; Scott et al., 1982; Williams et al., 1990), were also found resistant to FAW when 20 neonate larvae/plant released twice on 6- to 10-leaf plants. According to the study by Ni et al. (2011), two genotypes namely Mp708 and FAW 706 were found to be resistant based on foliar damage rating against FAW upon infestation with 15–20 neonates on 6-leaf stage plants, while Ab24E and EPM 6 were found the most susceptible. In another study, Ni et al. (2014) evaluated 20 maize lines from the USDA-ARS Germplasm Enhancement of Maize (GEM) Program for resistance to FAW. The study revealed that the entries with genetic backgrounds, UR11003:S0302, CUBA164-1, and DK7, that were derived from tropical maize germplasm lines originated from Uruguay, Cuba, and Thailand, respectively, were recorded as resistant based on leaf injury ratings. Abel et al. (2020) reported that lines derived from XL370A maize germplasm namely GEMN-0095, GEMN-0096, and GEMN-0133 were moderately resistant to FAW when infested with 25 ± 5 FAW neonates at V_6_-V_7_ stage. First-generation CIMMYT maize hybrids, CKIR06007, CKIR06001, CKDHL164288/CLRCY039, were reported as tolerant to FAW (Prasanna, 2018). CIMMYT lines namely CML 71, CML 124, CML 125, CML 333, CML 334, CML 338, CML 370, CML 372, and CML 574 were also reported as resistant to FAW (Prasanna et al., 2021) by releasing 5–10 neonate larvae or 20 black-head stage eggs at different nodes of maize plant at V_5_ stage. Several morphological and biochemical factors are responsible for imparting resistance to FAW in maize. Morphological traits, such as thicker cell wall complex of epidermal layer (Davis et al., 1995), leaf toughness (Bernal et al., 2015), and trichome density (Moya-Raygoza, 2016), were found to contribute to FAW resistance. The biochemical traits include crude and acid detergent fiber (Maynard, 1970; Hedin et al., 1990), aspartic acid and tyrosine (Hedin et al., 1990), polyphenol compound maysin ([2”-O-α-L-rhamnosyl-6-C-(6-deoxy-xylo-hexos-4-ulosyl)-luteolin] (Gueldner et al., 1991), chlorogenic acid, flavone C-glycoside (Maysin) (Snook et al., 1994), hemicellulose (Hedin et al., 1996; Williams et al., 1998), lignins (Hedin et al., 1996), cysteine proteinase (Jiang et al., 1995; Lopez et al., 2007), elevated defensive protein followed by insect herbivory, low P/C ratio (Chen et al., 2009), and benzoxazinoids (Glauser et al., 2011) were reported to confer FAW resistance in maize.

### FAW Resistance in Wild Ancestors

In the present study, efforts were also made to find resistance response in wild ancestor *Z. mays* ssp. *parviglumis* and the inbred line (MIL 1-11) derived by crossing with wild ancestors (Table 1). The wild ancestor screened in the present study showed promising results with minimum FAW damage (LDR-3.69). It could be due to their higher vigor, leaf shape, or inbuilt mechanisms of resistance. Similarly, there were reports on wild ancestors exhibiting resistance against other maize insect pests, including European corn borer and Asiatic corn borer, are available (Pasztor and Borsos, 1990; Ramirez, 1997). The previous studies indicated that morphological traits, such as leaf toughness and leaf trichome density in *Z.mays* ssp. *parviglumis* (Mammadov et al., 2018), contribute to resistance to FAW. Szczepaniec et al. (2012) reported that higher expression of herbivore resistance genes, wound inducible protein (wip1), maize protease inhibitor (mpi), pathogenesis-related protein (PR-1) in *Z. mays* ssp. *parviglumis* compared to normal maize lead to resistance against FAW herbivory. In *Z. mays diploperrennis*, the chemical composition of leaves, such as apimaysin and 3′-methoxymaysin in leaves or silks (Gueldner et al., 1992), caffeoylquinic acids and other luteolin derivates (Farias-Rivera et al., 2003), wip1, PR-1, chitinase gene, maysin, and chlorogenic acid (Szczepaniec et al., 2012) impart resistance to FAW. Further, the emission of herbivore-induced volatiles include indole and a large number of mono and sesquiterpenes emitted from FAW leaf herbivory attracts larval parasitoids (Mammadov et al., 2018). However, the leaf shape can also be considered as one of the mechanisms for imparting resistance to FAW as it is completely different in *Z. mays* ssp. *parviglumis* and does not have a broad leaf to feed by FAW. Another probable reason for resistance might be due to the faster growth of wild ancestors, and the FAW larvae may miss their preferable feeding stage (early whorl to mid whorl stages) leading to significant low damage (Azeez et al., 2018; Prasanna et al., 2018). Identification of wild ancestors of maize with resistance to FAW can provide a basis for their utilization in a breeding program (Doebley, 1990; Choudhary et al., 2017) and also develop FAW resistant lines. The present study would continue its efforts to identify novel resistant sources against FAW and also map the genomic regions determining resistance to find novel QTL(s)/genes.

## Conclusion

The present study indicated that the artificial infestation of maize genotypes with 20 FAW neonates at the V_5_ phenological stage is the most appropriate methodology for the characterization of maize germplasm. The results of screening showed that LDR (Leaf Damage Rating) depends on genotype, neonate counts, and days after infestation. Large-scale screening of maize germplasm against FAW can be done by comparing injury levels through this standardized screening technique. The identified genotypes, such as DMRE 63, DML 163-1, CML 71, CML 141, CML 337, CML 346, and the wild ancestor *Z. mays* ssp. *parviglumis*, can be used as the source of resistance to FAW. The findings from the study would result in the development of breeding schemes to utilize in developing durable resistance genotypes, decreased dependency on chemical insecticides, improved food and nutritional security, and enhancement of the resilience of resource-constrained smallholders.

## Data Availability Statement

The raw data supporting the conclusions of this article will be made available by the authors, without undue reservation.

## Author Contributions

PS: conceptualization, investigation, methodology, and draft writing. JS: project administration, supervision, and manuscript review. KY: germplasm maintenance, ensuring seed availability, and manuscript review. CK, KSR, SS, SJ, BK, KK, KS, JP, JV, and VK: manuscript review and editing. CK and AD: statistical analysis. SR: analysis, interpretation, and review of the manuscript. All authors read, corrected, and approved the manuscript.

## Funding

We sincerely thank the ICAR-National Agricultural Science Fund (NASF; competitive Grant No. NASF/ABAP(SM)-8001/2019-20) for funding this research and consistent support.

## Conflict of Interest

The authors declare that the research was conducted in the absence of any commercial or financial relationships that could be construed as a potential conflict of interest.

## Publisher's Note

All claims expressed in this article are solely those of the authors and do not necessarily represent those of their affiliated organizations, or those of the publisher, the editors and the reviewers. Any product that may be evaluated in this article, or claim that may be made by its manufacturer, is not guaranteed or endorsed by the publisher.
